# National Regulation on Processing Data for Scientific Research Purposes and Biobanking Activities: Reflections on the Experience in Austria

**DOI:** 10.1007/s41649-022-00231-4

**Published:** 2022-11-14

**Authors:** Joanna Osiejewicz, Dmytro M. Zherlitsyn, Svitlana M. Zadorozhna, Oleksii V. Tavolzhanskyi, Maryna O. Dei

**Affiliations:** 1 Department of International Legal Communication, University of Warsaw, Warsaw, Poland; 2 Department of the Economic Cybernetics, National University of Life and Environmental Sciences of Ukraine, Kiev, Ukraine; 3 Department of Procedural Law, Faculty of Law, Yuriy Fedkovych Chernivtsi National University, Chernivtsi, Ukraine; 4 Department of Criminology and Criminal and Executive Law, Yaroslav Mudryi National Law University, Kharkiv, Ukraine; 5 Department of Constitutional and Administrative Law, Faculty of Law, National Aviation University, Kiev, Ukraine

**Keywords:** Data governance, Biobank, Data protection, Austria, Poland, Ukraine, Data ethics

## Abstract

The application of the latest technologies in biology and medicine has brought them to a qualitatively new level of possibilities. Worldwide, biobanking is actively developing through the creation of biobanks of various types and purposes, whose resources are used to solve therapeutic or scientific problems. Legal science remains an open question concerning the boundary that runs between the right to data protection and the scope of disclosure of data needed for medical purposes. In this article, the author considers peculiarities of data processing in the context of biobanking activity on the example of Austria and its national legislation. In addition, the article reveals features of the approaches of the European Court of Human Rights (ECtHR) and the Council of Europe to the issue of biobanking in general, its characteristics in the context of data, and legal regulation of this phenomenon in the national law of states. The author devoted an important part of the study to the role of Austria’s experience in the context of data processing for scientific purposes and the development of biobanking for a number of other European states. The *aim* of the article is to analyze the Austrian legislation on data processing for scientific research and biobanking, the attitude of the Council of Europe to this phenomenon, and the practice of the ECtHR, as well as to consider the impact of the current world situation on these activities. The leading method of research used in the article is the formal-legal method. The article analyzes the Austrian law in the context of data processing in medical research, the relationship of the specifics of personal data protection, and the need to disclose them for scientific purposes. The author pays special attention to the influence of Austrian law on the legislation of other countries, which is reflected in the conclusions to the article. In addition, based on an analysis of the application of the Austrian experience to the legislation of Poland and Ukraine, the author points out the necessary changes that should be made in the laws of these countries.

## Introduction 

In European countries, biobanking has a history of more than 30 years. Biobanking is a system for collecting, processing, storing, and analyzing samples of biological materials and associated clinical data for scientific and biomedical research. The ongoing research in the field of medicine, particularly genetics, is unthinkable without biobanks, as well as the development and appearance on the market of new drugs, the development of personalized and regenerative medicine, and the introduction of innovative diagnostic tools into clinical practice. It can be argued that this branch of science is very important for the development of all medicine around the world. At the international level, this issue has been addressed several times at meetings of the World Medical Association. In 2002, the WMA Declaration on Ethical Considerations Regarding Health Databases was adopted. In 2016, it was revised and called the WMA Declaration of Taipei on Ethical Considerations Regarding Health Databases and Biobanks (WMA 2016). According to this declaration, confidentiality is essential for maintaining trust and integrity in Health Databases and Biobanks and the collection, storage, and use of data and biological material from individuals capable of giving consent must be voluntary. It also prescribes the rights of individuals to take certain actions in relation to their personal data, in particular: to request for and be provided with information about their data; to request corrections of mistakes; to alter their consent; or to ask for their identifiable data to be withdrawn from the Health Database. The declaration also prescribes the principles on which the regulation of Health Databases and Biobanks should be based, namely: protection of individuals, transparency, participation and inclusion, accountability. In addition to the well-recognized benefits of IPD sharing, one of the most widely discussed risks of individual data sharing is privacy risk. Especially since the implementation of the EU General Data Protection Regulation (GDPR 2016), the legal basis for the secondary use of clinical trial data has been discussed between the European Commission and the European Data Protection Commission. This regulation, which is specific to the EU, has a major impact on the world because the exchange of personal data of an individual from the EU with non-EU countries is governed by this regulation. Anonymized (anonymized) data is not subject to the GDPR. Genuine anonymized data may have more restrictions, and processing personal data into anonymized data before secondary use also requires a legal framework. A justification for the secondary use of pseudo-anonymized and/or coded personal data is mandatory. From a GDPR perspective, there are two possible justifications: (a) the application of GDPR Article 89 to scientific research allows for the waiver of an individual’s explicit consent, subject to appropriate privacy protections; (b) informed consent for secondary use under GDPR Recital 33, which can be interpreted as broad consent in accordance with “recognized ethical standards” (Kurihara et al. 2020; Lytvyn et al. 2021). The ethical principles emphasize that activities and research related to databases and biobanks should benefit society, particularly in the context of public health. Protection of privacy and confidentiality is essential to maintain trust, and physicians have both ethical and legal obligations to protect data provided by their patients. However, detailed rules regarding the data to be disclosed to individuals are listed in cases where data and materials are stored for multiple and unlimited use, and the type of consent to be obtained is not specified (Dhai 2016). The GDPR provides the data subjects with several rights, known as individual rights, but at the same time through Article 89 it enables two co-existing avenues of depriving the subjects of these rights if necessary for research: first, one that permits the researchers to invoke the GDPR norms directly for the purposes of a particular project; second, one that requires the Member State national law or EU law to be adopted so that derogations can take place (Slokenberga et al. 2021). 

So, European countries have already developed a system of sources of legal regulation in the sphere of biobanks; moreover, for the purpose of their successful development, a number of specialized organizations have been created (Sarmanaev et al. 2019). At the same time, there are two approaches to the legal regulation of the emerging relations in this area: the adoption of special acts dedicated exclusively to biobanks, or the inclusion of relevant provisions in the normative acts covering a wider range of relations arising in connection with genetic research. In addition, foreign countries possess not only “rich” biobanks, but also a legislative base in this sphere that has been developed over the years. For example, in the European Union, the activities of biobanks are subject to detailed legal regulation at the level of the EU and individual countries, as well as at the level of international organizations, such as the Council of Europe.

Based on the special legal capacity of a biobank, the wide range of persons cooperating with it, the determination of the system of possible risks of activities, and the lack of detailed rules of conduct, we formulate the basic principles of the human biological materials bank: respect for donor confidentiality and informing donors of the purposes of the biomaterials provided, ensuring the safety of bank employees who come into contact with biological materials, and the availability of biomaterial for scientific research under the condition that it is not used for research.

At the same time, the issue of personal data protection in the context of the functioning of biobanks remains important. The protection of personal data itself is very important and requires precise compliance, as it may violate fundamental human rights. At the same time, such disclosure may be necessary for medical purposes. The pertinent question remains: where are the boundaries of the need for compliance with personal data protection legislation and the need for disclosure for medical purposes. The purpose of this article is to analyze the Austrian legislation on data processing for research purposes and biobanking activities, the attitude of the Council of Europe to this phenomenon, and the practice of the European Court of Human Rights (ECtHR), as well as to consider the impact of the current world situation on these activities.

The new regulations of the Amendment Act have to promote positive development in Austria as a location for science, research, and innovation. Thus, the law provides for rules on pseudonymization and register research for archiving purposes in the public interest, scientific or historical research purposes, and statistical purposes. Existing registers can now be opened for scientific institutions if it is useful for the purpose of science and research in the field of life and social sciences. A major goal is to create legal certainty for existing sample and data collections, especially biobanks, as well as for other scientific archives (Lytvyn et al. 2022). The possibility of unlimited storage periods for relevant data is provided. Science and research should also not be hindered by the fact that personal data may not be processed for scientific research purposes. The legal framework ought to ensure appropriate freedom of movement and clear liability rules for science and research, so that clear distinctions between illegal and legally compliant behavior are possible.

Since Austria is a member state of leading international actors such as the Council of Europe, its law cannot be considered independent of the law of those organizations. Therefore, notwithstanding the fact that the article focuses on Austrian law, the author considers it appropriate to pay considerable attention also to European law in the context of the topic of the study. Thus, for example, the European General Data Protection Regulation (GDPR 2016, 1–88) required a number of adjustments in the Austrian law. The changes were undertaken with the Research Organization Act (2018). The new regulations, which were developed after consulting the stakeholders, were also intended to ensure an increase in data quality and the removal of bureaucratic obstacles for science, research, and statistics. The adjustments in the field of science and research came into force together with the so-called Material Data Protection Amendment Act (2018) and an associated Data Protection Deregulation Act (2018) on 25 May 2018. An internal evaluation is planned for 2023 (Parlamentskorrespondenz Nr 378, 2018).

## Methods

In this article, the author decided to use a system of philosophical, general scientific, and special methods, which ensure the reliability of the obtained results and the achievement of the formulated objectives of the article. The most commonly used method that the author applied to achieve the objectives of the article is the formal-legal method. Its use provided an opportunity not only to analyze the law of Austria, Ukraine, and Poland, as well as the norms of international law, but also to compare different legal norms governing the same issue in different jurisdictions. In addition, the formal-legal method was used when reviewing the practice of the ECtHR.

The use of the method of comparison provided an opportunity to identify how the same topical issues are regulated differently by participants in international relations and which ways are the most advantageous. In addition, the comparative method was used by the author in analyzing the concepts and their use in different norms. For example, the comparative method helped to identify differences between the legal regulation of biobanking in Austria, Ukraine, and Poland. Also, the method of comparison was used to identify inaccuracies in the legislation, which can be eliminated by applying Austrian practice.

Also in the process of research, the author applied methods of scientific cognition, which were used to study the features and characteristics of biobanking and its importance for the development of science and medicine in general. The results of scientific-cognitive activity with the use of the above method provided the need to clarify the ways to improve the legislation in the context of current trends of development and European standards, as well as taking into account the rule of law and respect for human rights.

Application of the systematic method allowed to generalize and systematize scattered data about biobanking and legal regulation of this issue. The method of analysis and synthesis made it possible to study theoretical information, international legal acts, legislation of the researched states, and on their basis to highlight the problems that exist in the studied sphere. This method was used for the general research of international law in the context of general regulation of biobanking by the acts of international law and those legal acts that regulate certain issues.

## Results

### Freedom of Science in Austria: A Legal Approach

In Austria, the absolute fundamental right to freedom of science is anchored in Art 17(1) StGG. Absolute means that it is a fundamental right that exists unless there is explicit legal reservation (Gamper and Kastelitz 2018). According to case law of the Austrian Constitutional Court (VfSlg 8136/1977, VfSlg 13978/1994), anyone who researches and teaches scientifically is entitled to scientific freedom. The object of freedom of research is to seek out new knowledge or to consolidate older knowledge in a certain field of knowledge (VfSlg 3191/1957). Even if the state may not subject the researcher to any specific, intentional restrictions to this freedom (VfSlg 8136/1977), the absolute fundamental rights are only guaranteed within the limits of general laws, that is within the immanent fundamental rights barriers (VfSlg 1777/1949, VfSlg 4732/1964, VfSlg 11737/1988, VfSlg 13978/1994). An encroachment on the freedom of science and teaching that prevents scientific activity (VfSlg 4881/1964) or even only restricts it (VfSlg 2823/1955) is only permissible if it is necessary and proportionate to protect another legal interest (VfSlg 2823/1955). It is therefore always necessary to weigh up the freedom of science and the legal interest protected by the interference (VfSlg 13978/1994). Fundamental rights clashes and conflicts that can arise in the area of scientific research with personal data must be resolved by achieving of balance between data protection and academic freedom, 22, i.e., the legal norm that has so far formed the most important data protection framework under simple law for scientific research and statistics (Gamper and Kastelitz 2018).

### Organization of Research

The GDPR (2016) offers the so-called multiple opening clauses, i.e., areas that can be regulated separately by the member states. It should be noted Article 9(4) GDPR, which stated Member States may maintain or introduce further conditions, including limitations, with regard to the processing of genetic data, biometric data, or data concerning health. This adjustment was made for Austria with the Data Protection Act (1999), the Research Organization Act (2018), and some other laws. A dedicated national law hence covers research in various domains detailing safeguard requirements.

Article 7 of the Data Protection Act (DPA) contains specific provisions for archiving purposes in the public interest, scientific or historical research purposes, or statistical purposes. From the wording of § 7 (1) DPA, it is clear that the standard regulates research purposes. However, only research projects that are based on Article 6 (1) (c) or (e) GDPR (2016, 1–88) as the legal basis are covered by Art. 7 DPA, while research purposes based on—predominantly or exclusively—private (commercial) interest are therefore not (Gamper and Kastelitz 2018, 121). In practical interpretation, however, it is likely to be more important that Art. 7 (1) DPA can apparently only refer to specific research projects that also have no personal results as their goal. That is why data collections for initially indefinite purposes or projects with personal results are subject to Art. 7 (2) DPA. Due to the indefinite term “determine” in Art. 7 (1) (2) DPA, there are also uncertainties as to whether the further processing of personal results from completed research projects is included if these have not been published.

In addition, the analysis of the provisions of the said Article 7 gives grounds to distinguish two groups of data processing for research purposes, to which different safeguards apply. The first one is the processing of personal data for scientific research purposes, which do not have as an ultimate goal to obtain personal results. In this case, the controller has the right to process the following data: publicly available, lawfully obtained for other research projects, and pseudonymized. The second group includes data whose processing is aimed at obtaining personal results. In this case, personal data may be processed only in the following cases: in accordance with special legal provisions, with the consent of the data subject, with and the approval of the Supervisory Authority. That is, the protection requirements depend on the purpose and the consequences for the data subjects (Kindt et al. 2021).

The Research Organization Act (ROA) regulates the general conditions for the processing of personal data for archiving purposes in the public interest, scientific or historical research purposes and for statistical purposes. This law created clarity about what is to be understood as a “public body” within the meaning of the GDPR. Further definitions for “open access,” “open data,” “big data,” “research material,” etc. can be found in Art. 2b (1)-(13) ROA. Article 2c ROA opened up the possibility of using area-specific personal identifiers for purposes of science and research. The use of these area-specific personal identifiers represents a pseudonymization within the meaning of Art. 4 (5) GDPR and can therefore be applied for the processing of special categories of personal data in the field of science and research. Article 2d contains the basic provisions for the protection of personal data. It requires particular measures to be taken.

Firstly, access to personal data that is processed with automation support on the basis of Sect. 1 of the ROA must be logged without gaps. Secondly, responsible persons and processors who process personal data on the basis of the Sect. 1 of the ROA and their employees have to keep secret personal data that have been entrusted to them exclusively on the basis of this section or that have become accessible, without prejudice to other statutory confidentiality obligations, unless there is a legally permissible reason for the transfer of the entrusted or accessible personal data (data secrecy). Thirdly, personal data that are processed with automated support on the basis of Sect. 1 of the ROA may only be processed for the purposes of the ROA. Natural persons whose personal data are processed on the basis of Sect. 1 of the ROA may not suffer any disadvantages from the processing, whereby the processing in accordance with this section does not constitute a disadvantage. Fourthly, those responsible who carry out processing on the basis of Art. 2d (2), i.e., who use area-specific personal identifiers, are obliged (a) to make publicly available data on the use of this legal basis on the Internet; (b) if their data is provided with area-specific personal identifiers, to delete the name information in any case; (c) to appoint a data protection officer (Art. 37 GDPR) in any case before using registers; (d) to expressly define the distribution of tasks in the processing of data (§ 2b (5)) between the organizational units and between employees; (e) to bind the processing of data to the existence of valid orders from the organizational units and employees authorized to issue orders; (f) to instruct every employee about their duties under this federal law and internal data protection regulations including data security regulations; (g) to regulate the access authorization to the rooms in which the processing of the data (§ 2b (5)) actually takes place; (h) to regulate the access authorization to data (§ 2b (5)) and programs and the protection of the data carrier against inspection and use by unauthorized persons; (i) to determine the authorization to operate the data processing equipment and to secure each device against unauthorized start-up by taking precautions on the machines or programs used; (j) to provide a documentation on measures taken according to lit. (d) to (i) in order to facilitate control and preservation of evidence; (k) to attach a declaration signed by the person authorized to dispose of the databases from which the personal data is to be determined to their request for the provision of data pursuant to Art. 2 (3).

The declaration should state that they provide the person responsible with the data for the investigation. Instead of this declaration, an enforcement title replacing this declaration (Sect. 367 (1) of the Enforcement Order, RGBl. No. 79/1896) can be submitted; (l) when processing data provided in accordance with Art. 2 (3) (Sect. 2b (5)), to assure that only the natural persons named in the application may access the data provided in accordance with Art. 2 (3); (m) if name details are transmitted in accordance with Art. 2 (3), to delete them after the purposes in accordance with Art. 89 (1) GDPR have been achieved.

According to § 2d (2.1) letter (a) to (d), research institutions may process all personal data in any case, in particular in the context of big data, personalized medicine, biomedical research, biobanks, and the transmission to other scientific institutions and contract processors, if (a) instead of the name, area-specific personal identifiers for the area of activity “research” or other unique identifiers are used for allocation or (b) the processing takes place in pseudonymized form (Art. 4 (5) GDPR) or c) publications take place (aa) not or (bb) only in anonymized or pseudonymized form or (cc) without names, addresses, or photos, or (d) the processing takes place exclusively for the purpose of anonymization or pseudonymization and there is no disclosure of directly personal data to third parties (Art. 4 (10) GDPR). It must therefore be ensured that direct personal data is not published under any circumstances.

Article 2d (2.3) is intended to place register-based research, which is expressly mentioned in Recital 157 of the GDPR, on a secure legal basis within Austria as well. Better research results can be obtained through the use of registers because they are based on a larger segment of the population. With this provision, all registers set up or operated by public bodies and authorities should be open to scientific institutions in the future. In this context, registers do not only mean publicly accessible registers within the meaning of Art. 3 (18) of the Federal Statistics Act (2000), but all directories, databases, or similar applications or processing platforms that are provided for by federal law. Public prosecution and criminal court registers as well as registers in the area of courts, lawyers, and notaries are excluded from this regulation. A reimbursement of costs has to be paid for the provision of register data (these costs are reimbursed to the public sector). The right to research registers exists regardless of whether the register in question contains personal data or not.^1^

In application of the ROA (Art. 2d (2.3.3.)), research institutions may process special categories of personal data if the person concerned voluntarily, in an informed manner, and unequivocally declares their will in the form of a declaration or other unambiguous confirmatory act, with the processing of the personal data concerning them to be in agreement. The purpose of the processing can be specified by providing a research area or several research areas, or a research project or parts of research projects (“broad consent”). The achievement of purposes according to Art. 89 (1) GDPR will probably be made impossible if the exercise of these rights would subsequently change research results (Rudiger 2020, 41). A serious impairment exists if the fulfillment of the obligations would involve a disproportionate effort for the person responsible (§ 2e ROA). For the purpose of quality management of research institutions, the person responsible may in particular process the following data directly on a personal basis, but only publish them in pseudonymized or anonymized form. The publication may occur, firstly, with regard to the persons who were or are active in teaching or research: all data according to Art. 2 g (1–4) ROA (all data that is relevant for the processing of funding); socio-biographical and socio-economic information; qualitative data, in particular regarding the relevance of the course for employment, the professional advancement and satisfaction, or the perception of the quality and relevance of their educational and training experience; quantitative data, in particular regarding the entry into professional life and further (training) education, income, type of contract, employment status, occupation, occupational status and activity (during the course), information on geographical and sectoral mobility (§ 2b (7)), and all academic functions, publications, third-party fundraising, and activities relating to technology transfer. Secondly, the publication may take place with regard to the persons who were or will be supervised within the framework of the teaching, the information mentioned, and quantitative data, in particular regarding study intensity, study method, qualification(s), obtained credit points, and subject of study.

To increase the transparency of processing in accordance with Art. 89 GDPR, scientific institutions (Art. 2b (12)) are allowed to enlist the scientific staff, who are in an upright employment relationship with the respective scientific institution, including a photo and a list of their publications, on a website of the scientific institution or in the context of publicly accessible reports of the scientific institution, unless the publication is likely to violate public safety, criminal justice, comprehensive national defense, foreign relations, or a legitimate private or business interest, whereby the publication of a photo according to lit. a can be objected to at any time. Alternatively, the scientific institutions are allowed to enlist the scientific staff, who are no longer in a regular employment relationship with the respective scientific institution, as well as students by name on a website of the scientific institution or in the context of publicly accessible reports from the scientific institution unless the publication is likely to harm public safety, the administration of justice, comprehensive national defense, foreign relations, or a legitimate private or business interest. Recipients of funding from federal funds, research contracts, and the like can be stored for at least 10 years for the purpose of establishing contact with names, personal characteristics, address and contact details, data on project partners, data on training, and data on funds received and mobility (Art. 9 (1) ROA). Key research areas and information on publications of former academic staff and students are allowed to be processed as well (Art. 2 g (1.3.) ROA). Apart from this, scientific institutions are allowed to process names, personal characteristics, and data on curriculum vitae of scientists and persons close to them (Art. 2 g (1.4.) ROA).

With regard to knowledge and technology transfer, Art. 2i stipulates that irrespective of any patent law provisions, processing, in particular within the meaning of Art. 2d (8) or the transmission of personal data, is permitted for technology transfer if this processing is necessary to maintain the functionality of the technology to be transferred. A cumulative condition is, in particular, to ensure through technology design in accordance with Art. 25 GDPR that third parties (Art. 4 (10) GDPR) have no actual knowledge of the transmitted data. Under these conditions, the rights of the data subject do not apply. Knowledge transfer is permitted under the conditions that area-specific personal identifiers are used in place of the name or that processing is carried out in pseudonymized form or there are no publications or publications take place only in anonymized or pseudonymized form/without name, address, photo, or processing is carried out solely for the purpose of anonymization or pseudonymization and there is no direct disclosure of personal data to third parties.

In the context of the study, it is also acceptable to consider two legal acts adopted in the framework of the EU, namely Directive (EU) 2019/1024 of the European Parliament and of the Council of 20 June 2019 on open data and the re-use of public sector information (recast) (EU 2019) and the Proposal for a Regulation of the European Parliament and of the Council on the European Health Data Space (European Commission 2022). Regarding the first Directive, we note that access to data is a fundamental right. The Charter of Fundamental Rights of the European Union (Charter) provides that everyone has the right to freedom of expression, including freedom to hold opinions and to receive and impart data and ideas without interference by public authority and regardless of frontiers. This Directive respects the fundamental rights and observes the principles recognized in particular by the Charter, including the right to privacy, the protection of personal data, the right to property, and the integration of persons with disabilities. The public sector authorities shall, by electronic means where possible and appropriate, process requests for reuse and make the document available for reuse to the applicant or, where a license is required, complete the offer of a license to the applicant within a reasonable time corresponding to the time limits set for processing requests for access to documents (EU 2019).

Regarding the Regulation, we emphasize that it notes that individuals today face difficulties in exercising their rights to their electronic health data, including access and transfer of their electronic health data domestically and abroad. The overall goal is for individuals in the EU to have greater control over their electronic health data in practice. It also aims to provide a legal framework consisting of robust EU and member state governance mechanisms as well as a secure processing environment. This will allow researchers, innovators, policy makers, and regulators at EU and Member State level to access relevant electronic health data to improve the diagnosis, treatment, and well-being of individuals, and to develop more effective and informed policies (European Commission 2022).

Thus, we can see that the Austrian legislation, based on the norms of European law, is actively developing the personal data protection sector in its various aspects. In addition, the adoption of the above-mentioned EU Regulation in 2022 allows us to assert that this issue continues to evolve at the level of member states as well, which indicates its relevance. In addition, we can state that with time Austrian law will also undergo changes, as the changes in the European legislation affect European countries in one way or another, and since the topic under study is very relevant today, we can assert that in the future the legal provisions in this sector will only evolve.

## Discussion

### Legal Regulation of Personal Data Protection and the Exertion in the Context of Biobanking in the Council of Europe and the European Court of Human Rights

The Committee of Ministers of the Council of Europe (2016) adopted Recommendation CM/Rec(2016)6 on research of biological materials of human origin. This document complements and improves the Recommendation REC(2006)4 of the Committee of Ministers to member states adopted earlier in 2006 on research using biological material of human origin (Council of Europe 2006).

Recommendation CM/Rec(2016)6 (Council of Europe 2016) takes into account new developments in biobanking, such as the increasingly diverse origins of biological materials held in collections, the difficulty of ensuring the non-identifiability of such samples, the increasing number of studies using materials from different collections, and the importance of research on biomaterials withdrawn from persons unable to give consent. The purpose of this Recommendation is to clarify and protect the fundamental rights of persons whose biological materials are intended for biomedical research. Their dignity, integrity, and privacy should be guaranteed, and at the same time, researchers should enjoy access to biological materials. This legal instrument establishes the conditions for obtaining and storing materials for future research, as well as their use in specific research projects, in particular concerning relevant information and the consent of the persons concerned. Thus, the Council of Europe (2016) drew its attention to the importance of the data issue in biobanking, emphasizing the importance of such activities for medicine in general.

In the context of the study, it is also important to note the Report of the Expert Group on Dealing with Ethical and Regulatory Challenges of International Biobank Research (Gottweis et al. 2012). This document emphasizes the role of biobanks, stating that they collect biological samples and related data for medical research and diagnostic purposes and systematize them for use by others. In Europe, the current vision is to integrate biobanks as part of a pan-European infrastructure to support medical research and health care. The long-term growth of medicine and life-saving science in Europe will depend on and be accelerated by these investments, which could potentially lead to innovations in medical research, drug development, and health care delivery. Nevertheless, the governance system for biobanking needs to be strengthened to adequately support the development of this new infrastructure.

We should also pay due attention to the practice of the ECtHR on this issue. Since the first major references to the Convention on Human Rights and Biomedicine, 1997 in two decisions handed down in 2004 (Glass v. The United Kingdom 2004; VO v. France 2004), the European Court of Human Rights has been called upon to deal with a growing number of cases involving various bioethical issues. Let us consider these cases in more detail. Thus, case No. 61827/00 Glass v. The United Kingdom (2004) illustrates some of the difficulties associated with concepts commonly used by lawyers and ethicists, such as “best interests,” “double effect,” and “futility.” Under the circumstances of the case, the applicants are a child (the first applicant) with severe mental and physical disabilities who requires 24 hour attention and his mother (the second applicant). The child was hospitalized several times. On one of those occasions, on 8 September 1998, the doctors discussed with the second applicant the use of morphine to relieve his suffering. The second applicant expressed her opposition to the use of morphine or other drugs to relieve stress. She told the doctors that if the first petitioner’s heart stopped, she would be expected to undergo resuscitation, including intubation. Dr Walker felt that this was not in the first petitioner’s best interest and stated that if death was imminent, all that could be offered was “morphine and tender and loving care.” Subsequently, the second complainant expressed a desire to take the first complainant home if the doctors were correct in believing that he was dying. The police officer present informed her that if she tried to remove him, she would be arrested. The hospital also indicated that if the family members present did not allow the doctors to begin administering diamorphine, the police would also remove them. After administering the drug, the child got worse. His relatives thought the deterioration was caused by the doctors’ actions and beat them up. After that, the doctors were unable to fulfill their obligations for some time. The General Medical Council concluded that its investigation showed that the doctors involved were not guilty of serious professional misconduct or serious misconduct and that the appealed treatment was justified in light of the emergency faced by the doctors at the hospital. The first complainant’s condition improved and he was taken home, but the doctors stated that they would no longer be able to provide care to the complainants. The plaintiffs filed a lawsuit claiming a violation of Art. 8 of the ECHR Right to Privacy and Family Life. After reviewing the case, the court ruled in favor of the plaintiff and acknowledged the existing violations.

In case No. 53924/00 VO v. France (2004), we note that it arose out of an incident involving a French doctor who mistakenly ruptured the amniotic sac of a pregnant woman, mistaking her for another patient who was not pregnant. The complainant brought the case to the European Court of Human Rights, charging that the French government’s failure to provide criminal penalties for accidentally destroying a fetus was incompatible with the state’s duty to protect the right to life of the unborn child under Article 2 Right to life. The application argued that the European Convention for the Protection of Human Rights and Fundamental Freedoms does not protect the rights of the unborn fetus. It also argued that recognizing the right to life to the unborn fetus would endanger the human rights of women by allowing the government to prioritize the rights of the fetus over those of the pregnant woman. In July 2004, the ECtHR issued a judgment in which it refused to recognize the fetus as a person under the European Convention.

Thus, ECtHR has considered and ruled on very sensitive issues under Articles 2, 3, 5, 6, and most often Article 8 of the European Convention on Human Rights, such as consent to treatment, examinations, or removal of organs and tissue; reproductive rights (prenatal diagnosis, artificial insemination, and the right to legal abortion); decisions in end of life situations; preservation of biological data by authorities; and the right to know one’s biological identity.

### The Importance of the Austrian Experience in the Context of the Legal Regulation of Data Processing for Scientific Research and Biobanks for Poland and Ukraine

The analysis of Austrian law in the context of data rights in biobanking can serve as an important and significant experience for other European countries. In the context of recent world events, first of all the armed aggression of the Russian Federation, the rallying of most European countries around one state requiring assistance in an armed conflict has been actively manifested. Thus, it became clear that Ukraine can become a new member of the European Union, and thus share European norms, standards, and principles, including in the context of biobanking. As of today, Ukrainian legislation has a rather limited range of normative legal acts, in one way or another, concerning the activity of biobanks. Such normative legal acts include Civil Code of Ukraine (2003), in particular Art. 290, which enshrines the human right to donate blood and its components, organs, other anatomical materials, and reproductive cells (Civil Code of Ukraine 2003; Law of Ukraine 1992) and several other laws. In spite of this, the law of Ukraine has insufficiently regulated the issue of the studied activity. Thus, the analysis of normative acts and local acts of health care organizations allows us to conclude that there is no systematic approach of the legislator to the formation of legislation on human biological materials banks. We believe that a law on biobanks and their activities or a general law on the legal regulation of the use of genomic technologies, including a section on the status of biobanks, should be developed.

The new statutory act should provide for the order of establishment, reorganization, and liquidation of a biobank, principles of its activity, specifics of its management, legal regime of property, and rules of relationships between donors and biobanks, and also pay special attention to the protection of the right to data and limits of this right in the implementation of medical projects. In order to develop such a legal act, it is worth taking into account not only the experience of the EU and international organizations, but also the peculiarities of Austrian law discussed in this article.

Another state for which the experience of Austria can serve as a good legal basis is Poland. Since both states are EU member states, their legal systems have some connection in those aspects regulated by the Union, including applying certain aspects of personal data protection issues in their legislation. Therefore, it is important to examine Polish law for the existence of legislation on biobanking. Polish law does not provide legislative rules for the correct operation of biobanks. To ensure the best standards for obtaining, storing, and working with biological material, it is crucial to ensure the harmonization of biobanks (Pawlikowski et al. 2010). In addition, the field of biobanking entails very different risks for participants than in the case of medical experimentation. Regarding biobanking in law, European and American doctrine indicates that these threats are related to the privacy and autonomy of donors, but in no way to life and health (Krekora-Zając 2019). Freedom of scientific research is one of the fundamental human and civil rights protected directly in the Polish Constitution. According to Article 73 of the Constitution, everyone is guaranteed freedom of artistic creativity, scientific research and the publication of their results, freedom of teaching, and freedom of use of cultural values. It is noted that this freedom is most broadly related to the receipt and dissemination of data also in the public interest (Constitution of the Republic of Poland 1997). This means that in other situations, the Constitution establishes the primacy of freedom of scientific research in such a way that, where the legal order does not set clear limits and consequences of their violation, scientific research is permissible, and the legislature, public administration, and all other actors are obliged to refrain from interfering with this freedom.

However, the researcher should always adhere in his work to the ethical principles derived from codes of good practice and soft law regulations. Above all, he cannot allow a situation in which the interests of society or scientific goals prevail over the good of the individual. Scientific progress must be made with respect for the dignity of each individual, which also extends to respect for the body and its parts, even after death. The researcher must take care to preserve the confidentiality of all data that might pose a direct or indirect risk to the deceased or his relatives. It is also a recognized ethical standard in scientific research that the human body or its parts cannot, in and of themselves, serve as a source of financial gain. Researchers and people who accumulate human biological material must be characterized by an attitude free of stigma and discrimination toward donors of material, their families, and persons belonging to a particular ethnic group. Activities for the collection and storage of biological material should be performed by people with knowledge and experience in the collection, processing, and storage of biological material, as well as awareness of the legal and ethical aspects related to the collection and long-term storage of biological material and human data.

Note also that the issue of the importance of Austrian legislation for Poland and Ukraine is also revealed in the context of international cooperation. International cooperation of biobanks facilitates creation of large collections, exchange of samples, multiple testing of the same samples, and comparative studies in geographical cohorts. Already existing Polish biobanks are willing to cooperate nationally and internationally. Thus, the experience of other states will be positive for the realization of such cooperation and for the development of this direction in the states as a whole.

## Conclusions

So, biobanks have become an important tool in the health care system, and their number and types continue to grow year after year. In data protection law, the so-called prohibition with reservation of permission applies. Accordingly, the processing of personal data is fundamentally prohibited and only permitted in those cases that are expressly mentioned in the law. It is well known that the fundamental right to the protection of personal data is not an unrestricted right. Regardless of the fact that processing of personal data is only permitted if one of the permissions applies, the data protection requirements described above must also be complied with.

Acts of international law also pay special attention to the issue of biobanking development and data protection, as well as the boundaries between information rights and medical and scientific research. The long-term growth of medicine and life-saving science in Europe will depend on and be accelerated by these investments, which have the potential to lead to innovation in medical research, drug development, and health care delivery. Dignity, integrity, and privacy must be guaranteed, and at the same time researchers must enjoy access to biological materials.

The experience of Austria is also important for the development of this direction in other states. So, for example, talking about Ukraine, it is worth noting that in Ukraine, it is necessary to adopt a separate legal act, comprehensively regulating the activities of biobanks. For a clear perception and understanding of the role of biobanks, the term biobank should be introduced into the Ukrainian legislation, fixing its deep, broadened definition. The activities of biobanks as repositories of special purposes have specific functions, which should be taken into account when formulating the definition of the term biobank and the regulatory framework for their activities. The appearance of the term biobank in legal acts will adapt the national legislation to the established conceptual apparatus and make it understandable to a wide range of people.

For Polish legislation, the experience of Austria will also be important in the context of the development of the state under the influence of European norms and standards. In addition, foreign experience is very important in the context of further international cooperation in the matter of biobanking, which can significantly develop all branches of medicine.

The main drawback of these acts is that they do not reflect the specifics of the legal regime of biobanks; in particular, they do not establish the procedure for the creation and termination of their activities, and the rights and obligations of the founders of biobanks, and do not address other issues. Meanwhile, the resolution of these issues is of important practical importance, primarily to protect the rights of subjects who have provided their biological material, respectively, data about themselves.

## Data Availability

Data will be available on request.

## Footnotes

1 p. 34, https://www.parlament.gv.at/PAKT/VHG/XXVI/I/I_00068/fname_686447.pdf.
