# Editorial Expression of Concern: National Regulation on Processing Data for Scientific Research Purposes and Biobanking Activities: Reflections on the Experience in Austria

**DOI:** 10.1007/s41649-024-00346-w

**Published:** 2024-12-09

**Authors:** Joanna Osiejewicz, Dmytro M. Zherlitsyn, Svitlana M. Zadorozhna, Oleksii V. Tavolzhanskyi, Maryna O. Dei

**Affiliations:** 1 Department of International Legal Communication, University of Warsaw, Warsaw, Poland; 2 Department of the Economic Cybernetics, National University of Life and Environmental Sciences of Ukraine, Kiev, Ukraine; 3 Department of Procedural Law, Faculty of Law, Yuriy Fedkovych Chernivtsi National University, Chernivtsi, Ukraine; 4 Department of Criminology and Criminal and Executive Law, Yaroslav Mudryi National Law University, Kharkiv, Ukraine; 5 Department of Constitutional and Administrative Law, Faculty of Law, National Aviation University, Kiev, Ukraine

**Editorial Expression of Concern: Asian Bioethics Review (2024) 16(1):47–63**

10.1007/s41649-022-00231-4

The Editor in Chief is issuing this Expression of Concern because an investigation by the publisher found evidence that the authors may have used a third party to submit this manuscript. The identities of the peer reviewers suggested by the authors at submission could not be verified. The authors have not been able to provide a satisfactory explanation of why these reviewers were suggested. These suggested peer reviewers were not selected, and the article has undergone peer review in line with the journal’s editorial policies. Given the irregularities in the submission process, readers are urged to interpret the results and conclusions of this article with caution. The authors disagree with this Expression of Concern.

